# Cardiovascular reflex contributions to sympathetic inhibition during low intensity dynamic leg exercise in healthy middle‐age

**DOI:** 10.14814/phy2.15821

**Published:** 2023-09-13

**Authors:** Catherine F. Notarius, Mark B. Badrov, Tomoyuki Tobushi, Daniel A. Keir, Evan Keys, John S. Floras

**Affiliations:** ^1^ University Health Network and Sinai Health Division of Cardiology Toronto General Research Institute Toronto Ontario Canada; ^2^ Faculty of Kinesiology and Physical Education University of Toronto Toronto Ontario Canada; ^3^ School of Kinesiology The University of Western Ontario London Ontario Canada; ^4^ Department of Medicine University of Toronto Toronto Ontario Canada

**Keywords:** aging, arterial chemoreflex, cardiopulmonary baroreflex, dynamic exercise, microneurography, muscle metaboreflex

## Abstract

Aging augments resting muscle sympathetic nerve activity (MSNA) and sympatho‐inhibition during mild dynamic 1‐leg exercise. To elucidate which reflexes elicit exercise‐induced inhibition, we recruited 19 (9 men) healthy volunteers (mean age 56 ± 9 SD years), assessed their peak oxygen uptake (VO_2peak_), and, on another day, measured heart rate (HR), blood pressure (BP) and MSNA (microneurography) at rest and during 1‐leg cycling (2 min each at 0 load and 30%–40% VO_2peak_), 3 times: (1) seated +2 min of postexercise circulatory occlusion (PECO) (elicit muscle metaboreflex); (2) supine (stimulate cardiopulmonary baroreflexes);and (3) seated, breathing 32% oxygen (suppress peripheral chemoreceptor reflex). While seated, MSNA decreased similarly during mild and moderate exercise (*p* < 0.001) with no increase during PECO (*p* = 0.44). Supine posture lowered resting MSNA (main effect *p* = 0.01) BP and HR. MSNA fell further (*p* = 0.04) along with diastolic BP and HR during mild, not moderate, supine cycling. Hyperoxia attenuated resting (main effect *p* = 0.01), but not exercise MSNA. In healthy middle‐age, the cardiopulmonary baroreflex and arterial chemoreflex modulate resting MSNA, but contrary to previous observations in young subjects, without counter‐regulatory offset by the sympatho‐excitatory metaboreflex, resulting in an augmented sympatho‐inhibitory response to mild dynamic leg exercise.

## 1 INTRODUCTION

Profound hemodynamic adjustments during dynamic exercise match oxygen supply to metabolic needs of active skeletal muscle. Largely under the control of the autonomic nervous system, these include increases in cardiac output, arterial blood pressure (BP), and skeletal muscle blood flow (Andersen & Saltin, 1985). In healthy individuals, leg and arm efferent sympathetic vasoconstrictor discharge alter rapidly and differentially, according to exercise mode (Saito & Mano, 1991) and intensity (Ichinose et al., 2008; Katayama et al., 2011), to facilitate such changes. In the healthy young, the pumping action of contracting muscle increases central blood volume, stimulating the sympatho‐inhibitory cardiopulmonary baroreflex. As a consequence, mild intensity dynamic leg exercise elicits a reflexive decrease in muscle sympathetic nerve activity (MSNA) (Katayama et al., 2014; Notarius, Millar, Doherty, et al., 2019; Ray et al., 1993) which, in the young, can be counteracted by stimulation of both the peripheral chemoreflex (inhaled 12% O_2_) (Katayama et al., 2016) and by the muscle metaboreflex (high intensity ischemic handgrip) (Katayama et al., 2020).

In prior work, we documented greater inhibition of MSNA burst incidence during mild 1‐leg cycling in healthy older than young adults. We hypothesized that such augmentation signaled an effect of age on reflexive responses to dynamic exercise (Notarius, Millar, Doherty, et al., 2019). Although cardiovascular reflexes were not evaluated in that protocol, we noted a 2‐fold greater rise in systolic BP in the older participants, and posited amplified arterial baroreflex‐mediated sympatho‐inhibition as one potential explanation for this age‐related finding (Fisher et al., 2010; Notarius, Millar, Doherty, et al., 2019).

With previous investigations describing, with age, an impaired sympatho‐excitatory muscle metaboreflex (some but not all studies [Houssiere, Najem, Pathak, et al., 2006; Markel et al., 2003]), attenuation of the sympatho‐excitatory peripheral chemoreflex (Davy et al., 1997); and augmentation of the sympathoinhibitory cardiopulmonary baroreflex (Davy et al., 1998), we designed the present protocol to evaluate the effect of each on muscle sympathetic firing rates of middle‐aged healthy subjects during dynamic 1‐leg exercise. We assessed the muscle metaboreflex by post‐exercise circulatory occlusion (PECO); the cardiopulmonary baroreflex by assuming a supine posture; and tonic peripheral chemoreflex input by administering 32% inspired oxygen (Lloyd et al., 1957). Our aims were to determine whether the leg muscle metaboreflex is activated by low intensity dynamic 1‐leg exercise in middle‐aged subjects, and to test the hypotheses that interventions involving cardiopulmonary baroreflex stimulation or peripheral chemoreflex suppression would augment the sympathetic inhibition evoked by such cycling.

## 2 METHODS

### 2.1 Subjects

Nineteen healthy medication‐free volunteers (9 men and 10 post‐menopausal women) were recruited through local advertisement and screened by medical history. Their mean age was 56 ± 9 years (mean ± SD; range 45 to 75). Mean height was 169 ± 10 cm; body weight was 71 ± 11 kg and body mass index (BMI) was 25 ± 3 kg/m^2^. Subjects were active recreationally but untrained. Since reflex sympathetic response may vary according to fitness status in older subjects (DeLorey, 2021; Notarius et al., 2012), sedentary individuals were excluded.

This study complies with the Declaration of Helsinki and was approved by the Research Ethics Boards of University Health Network (16–5381), and Mount Sinai Hospital (17‐0047‐E). Informed written consent was obtained from all participants.

### 2.2 Procedures and protocol

Prior to the laboratory study day, peak oxygen uptake (V̇O_2peak_) was assessed by open circuit spirometry (Quark CPET system, Cosmed USA Inc.) measured during an exhaustive ramp‐incremental exercise cycling test (17 watts/min). Briefly, subjects exercised until they could no longer maintain pedal speed above 50 rpm and the respiratory exchange ratio (VCO_2_/VO_2_) exceeded 1.1. The VO_2peak_ was expressed both as mL/kg⋅min^−1^ and as percent of the age, sex, body weight, and height‐predicted VO_2peak_ (Jones et al., 1985).

The experimental protocol was conducted in a quiet temperature‐controlled laboratory following 12 h of caffeine abstinence and 2 h after any food intake. Subjects were seated, with the right leg supported, while the left foot was secured to the pedal of a cycle ergometer (Monark Rehab Ergometer Trainer 881). Right upper arm BP was acquired and recorded automatically every minute (Dinamap Pro 100, Critikon). Heart rate (HR) was determined from lead II of an electrocardiogram. A respiratory belt was placed around the abdomen to monitor breathing.

After 10 min of quiet rest, baseline signals were acquired while seated during spontaneous breathing on a Powerlab platform (ADInstruments). We recorded HR, BP, and MSNA (microneurography; right fibular nerve) at rest and throughout left leg cycling for 4 min (2 min at zero load [unloaded or mild intensity] then 2 min at 30%–40% of the work rate [loaded or moderate intensity] at V̇O_2peak_, but halved, since only 1 leg exercised), during 3 reflex‐modulating interventions which were randomly assigned: (1) *Muscle Metaboreflex*: exercise seated while breathing room air, followed by 2 min of PECO achieved by inflating an upper leg cuff to 200 mmHg upon cessation of exercise followed by 2 min recovery after cuff deflation. This isolated the muscle metaboreflex, which is activated by stimulation of metabolically‐sensitive muscle afferents from the mechanics of muscle contraction (mechanoreflex), and from the sympathoexcitatory influence of volitional effort (central command); (2) *Peripheral Chemoreflex*: subjects were fitted with a mouthpiece and nose‐clip and the same sequence of rest and exercise and recovery was repeated while seated breathing hyperoxic air (32% oxygen), sufficient to inhibit the arterial chemoreceptor (Lloyd et al., 1957); (3) *Cardiopulmonary Baroreflex*: After assuming the supine posture and again breathing room air, the same pre‐cycle, 4 min cycling at 2 work rates, and recovery was repeated. After each intervention, subjects were asked to quantify their effort using a 0–10 rating of perceived exertion scale (RPE) during each exercise intensity.

### 2.3 Microneurography

Multiunit recordings of post‐ganglionic MSNA were obtained with a unipolar tungsten electrode inserted selectively into a muscle‐nerve fascicle of the right peroneal (fibular) nerve, posterior to the fibular head. The raw neurogram was amplified by 20,000–50,000 (nerve traffic analyzer, model 662C‐3, University of Iowa), filtered at a bandwidth of 700–2000 Hz, rectified and integrated (time constant 0.1 s) to obtain a mean voltage display of MSNA. Recordings were quantified using a customized analytic program based on a LabVIEW® (National Instruments) platform, as previously described (Notarius et al., 1999). MSNA was expressed both as burst frequency (bursts/min) and, to account for changes in HR, burst incidence (bursts/100 heart beats), but not as total MSNA, due to electrode displacement during exercise, obliging occasional need to re‐establish a high‐quality recording by electrode site adjustment.

### 2.4 Statistical analysis

Data are presented as mean ± standard error. Baseline dependent measures were compared across interventions by a one‐way repeated measures analysis of variance (ANOVA) with individual post‐hoc comparisons using a Student Newman–Keuls (SNK) test (SigmaStat™ for Windows, Ver. 3.5, Systat Software Inc.). Two‐way ANOVA was used to assess between‐ intervention (seated normoxia, seated hyperoxia, supine) differences in dependent variables over time (pre cycle, zero load cycling, moderate load cycling exercise). A post‐hoc SNK test was applied to assess individual differences between means. The muscle metaboreflex was assessed by one‐way repeated measures ANOVA with post‐hoc comparison of the second minute of PECO to the second minute of the loaded exercise intensity. To test for a systematic difference in responses between males and females, dependent measures were compared with a two‐way ANOVA with group and time as main effects for each intervention separately and individual post‐hoc comparisons were made using a SNK.

## 3 RESULTS

Above average exercise capacity was confirmed in all subjects. They had a mean (±SE) V̇O_2peak_ of 32.5 ± 2.3 mL/kg⋅min^−1^, which was equivalent to 120 ± 6% of predicted. Stable MSNA nerve sites were achieved in all, but with some electrode re‐adjustment necessary during exercise in most participants.

**FIGURE 1:**
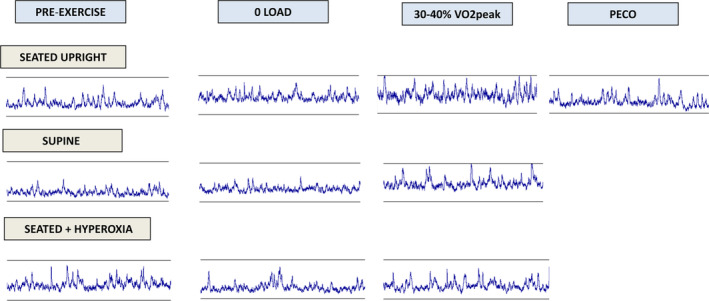
Representative 30 s MSNA microneurographic tracings in one subject before and during mild (0 load) and moderate (30%–40% VO2peak) one‐leg exercise while cycling(1) seated with PECO (top traces); (2) supine (center traces); and (3) seated with hyperoxia (lower traces).

Subjects' rating of perceived exertion (RPE) on the modified Borg scale (0–10) during mild unloaded (1.5 ± 0.2) and moderate loaded exercise (3 ± 0.2) were identical among the three interventions (*p* = 0.5) confirming similar relative exercise intensities. Figure 1 shows representative MSNA recordings at rest, during mild unloaded and moderate loaded one‐leg cycling exercise while: seated followed by PECO; in the supine posture; seated while breathing 32% oxygen.

### 3.1 Effect of reflex interventions during exercise

#### 3.1.1 Muscle metaboreflex

**FIGURE 2:**
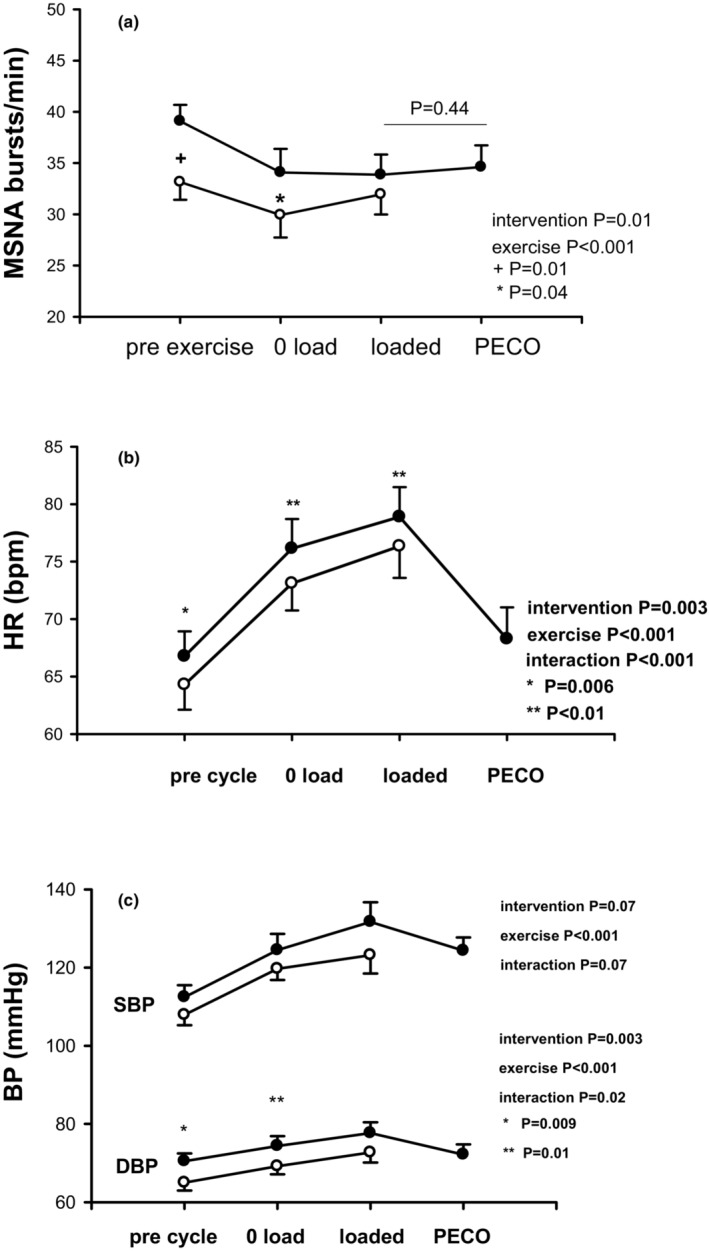
(a) MSNA burst frequency is significantly lower during supine rest and mild exercise (open circles) compared with seated with no increase during PECO (closed circles). (b) Heart rate is significantly lower at rest and during supine exercise at both intensities than seated exercise with no effect of PECO. (c) There is no difference in systolic blood pressure due to supine posture at rest or exercise, but diastolic blood pressure is significantly lower at rest and during mild exercise while supine. Group means (±SE). The main effects and interactions listed include all three interventions, which are graphed separately in Figures 2 and 3 for clarity.

While cycling seated, both MSNA burst frequency and burst incidence decreased similarly at mild and moderate intensities (*p* < 0.001) and HR and systolic and diastolic BP increased progressively, as expected (*p* < 0.001). MSNA did not rebound with PECO at termination of exercise (*p* = 0.44, vs. loaded cycle) (Figure 2).

#### 3.1.2 Cardiopulmonary baroreflex

Compared to sitting, supine posture lowered resting MSNA burst frequency and incidence (*p* = 0.01 and *p* = 0.003), systolic and diastolic BP, and HR (*p* < 0.001) (Table 1). MSNA fell further (*p* = 0.04 vs. seated) during mild (*p* = 0.01), but not moderate, supine cycling (Figure 2a) as did diastolic BP (Figure 2c) whereas the exercise systolic BP was unchanged. Supine posture attenuated the rise in HR during exercise at both intensities (*p* < 0.01 vs. seated) (Figure 2b).

**TABLE 1:** Baseline pre‐exercise Data. (mean ± S.E.).

	Seated	Supine	Hyperoxia	*p*‐Value
HR (bpm)	67 ± 2	64 ± 2*†	68 ± 2	<0.001
SBP (mmHg)	112 ± 3	108 ± 3*†	116 ± 3*	<0.001
DBP (mmHg)	70 ± 2	65 ± 2*†	73 ± 3	<0.001
MSNA (b/min)	39 ± 2	33 ± 2*	35 ± 2*	<0.001
MSNA (b/100HB)	59 ± 3	52 ± 3*	52 ± 3*	<0.001

*Note*: One‐way ANOVA for repeated measures revealed that heart rate and blood pressure pre exercise are lowest during supine posture with only systolic blood pressure highest when seated breathing hyperoxic air compared with normoxic air. Both MSNA burst frequency and incidence are lower while either supine or seated with hyperoxia compared with seated room air. *, versus seated room air; †, supine versus hyperoxia.

#### 3.1.3 Peripheral chemoreflex

**FIGURE 3:**
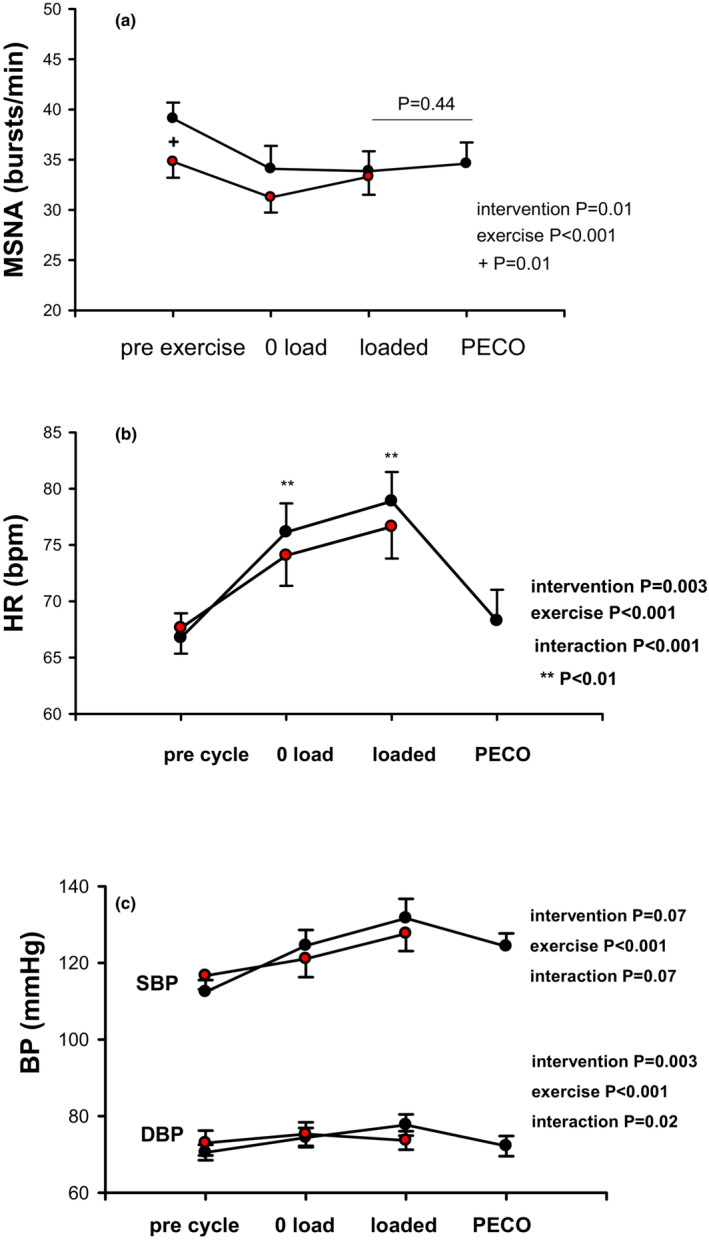
(a) Breathing 32% oxygen (hyperoxia) decreases MSNA burst frequency at rest but not during exercise (red circles). (b) Heart rate during hyperoxic breathing is similar at rest but significantly lower during both exercise intensities compared with breathing room air (black circles). (c) Systolic and diastolic blood pressure before and during exercise are both unchanged during hyperoxic breathing compared with room air.

Hyperoxia also attenuated resting MSNA burst frequency and incidence (*p* = 0.01 and *p* = 0.003), but in contrast had no effect on exercise responses (Figure 3a). Resting HR while breathing 32% oxygen was unchanged from seated normoxia but the increase during exercise was significantly blunted during both cycling intensities (*p* = 0.01; interaction *p* < 0.001) (Table 1) (Figure 3b). Resting systolic BP was significantly higher while breathing hyperoxic air compared with seated normoxia (*p* = 0.03) (Table 1). However, BP responses during exercise did not differ (Figure 3c).

#### 3.1.4 Sex differences

A sub‐analysis comparing responses in the nine males versus 10 females showed no sex difference in reflex contributions to sympatho‐inhibition during exercise although baseline MSNA burst incidence was lower in females than males (*p* = 0.008). Systolic BP in males was significantly higher at rest during seated hyperoxia versus supine and seated room air conditions (*p* = 0.001) but not in females (*p* = 0.157) (data not shown).

## 4 DISCUSSION

When recorded at rest in these healthy middle‐aged participants, prior to exercise, both supine posture and breathing 32% O_2_ reduced MSNA burst frequency and incidence relative to values obtained seated, when breathing room air. The principal sympatho‐neural adaptations to 1‐leg cycling observed were: (1) the absence of leg‐muscle metaboreflex‐induced sympathetic excitation and; (2) a greater fall in MSNA during supine, relative to seated, exercise at a mild but not at a moderate workload; (3) no further change in MSNA during exercise when breathing hyperoxic air.

### 4.1 Muscle metaboreflex

Previous work involving young, primarily male, volunteers revealed that at exercise intensities greater than 40% of V̇O_2peak_, the reflex decrease in MSNA can be overridden by the muscle metaboreflex, resulting in a progressive increase in MSNA (Katayama et al., 2020). Even during low intensity leg cycling (10–20 watts), the drop in MSNA could be blunted by simultaneous activation of the muscle metaboreflex in the arm (Katayama et al., 2020). Prior studies involving static and dynamic handgrip exercise reported age‐related diminution of the efferent sympathetic response to muscle metaboreflex stimulation (Houssiere, Najem, Pathak, et al., 2006; Markel et al., 2003). In the present study, when thigh PECO was applied after 4 min of low intensity cycling, BP remained elevated, but MSNA, which would have increased had the metaboreflex been stimulated, was unchanged. Of note, prior literature involving young healthy subjects, studied seated, reported an increase in MSNA only when the arm circulation was occluded after moderate leg or arm exercise matched for intensity (Ray et al., 1992). A recent report also showed that the muscle metaboreflex was less likely to be engaged if moderate intensity exercise was performed when seated (Joshi & Edgell, 2019). In that report, Joshi and Edgell commented that activation of the arterial baroreceptor while seated could “brake” the metaboreflex.

### 4.2 Cardiopulmonary baroreflexes

In healthy young subjects, MSNA, recorded from the median nerve, rises during mild, and moderate isometric leg exercise yet falls during dynamic leg exercise at the same intensities (Saito & Mano, 1991), as does MSNA recorded from the contra‐lateral fibular nerve during 1‐leg knee extension, in parallel with an increase in central venous pressure (Ray et al., 1993). A fall in median nerve MSNA also has been observed during graded 2‐leg cycling but only in young healthy subjects and at work rates less than 40% of V̇O_2peak_ (Ichinose et al., 2008; Katayama et al., 2011). In a study by Katayama et al, cycling at 60 rpm had no effect on median nerve MSNA, but increasing pedal rates by 33% raised stroke volume and cardiac output and reduced MSNA significantly (Katayama et al., 2014). Activation of the sympatho‐inhibitory cardiopulmonary baroreflexes, as a consequence of a rostral shift in blood volume due to activation of the leg‐muscle pump during seated low‐intensity exercise, was invoked to explain these observations (Ray et al., 1993; Katayama et al., 2014). However, when dynamic leg extension was performed supine, central venous pressure did not rise further and MSNA did not fall (Ray et al., 1993).

In a recent report, central venous pressure during a similar mild dynamic leg exercise protocol did not differ between older and young healthy subjects (Katayama et al., 2022). Assuming similar engagement of the leg muscle pump at low‐intensity exercise (Shiotani et al., 2002), the prior observation that inhibitory cardiopulmonary reflex modulation of MSNA is augmented with age (Davy et al., 1998) could account for the greater sympatho‐inhibition during mild and moderate cycling we observed previously in healthy middle‐aged compared with young subjects (Notarius, Millar, Doherty, et al., 2019). In our present cohort of middle‐aged participants, MSNA burst frequency decreased and remained significantly lower while supine, but only at mild cycling intensity. Sympatho‐inhibition was not observed at moderate intensity cycling, possibly due to concurrent activation of sympatho‐excitatory mechano‐ and metabo‐reflexes, which appear more potent when assessed in the supine rather than upright posture (Joshi & Edgell, 2019). Our current finding is unique to middle‐age healthy subjects and in direct contrast to early work in young men where no further change in MSNA during supine exercise was observed after an initial decrease at rest (Ray et al., 1993).

### 4.3 Peripheral chemoreflex

Changes in MSNA during leg exercise in healthy subjects depend not only on their age (Notarius, Millar, Doherty, et al., 2019), the type of exercise employed (static or dynamic) (Saito & Mano, 1991), the intensity (Ichinose et al., 2008; Katayama et al., 2011) and duration of exercise (Saito et al., 1997), but also on the oxygen concentration of inspired air (Katayama et al., 2011). In a canine model, in which hind‐limb peak conductance during carotid chemoreceptor inhibition by hyperoxia was compared to that with alpha‐adrenergic blockade, the peripheral arterial chemoreflex was judged responsible for approximately 30% of tonic sympathetic vasoconstrictor outflow during exercise (Stickland et al., 2007). In young men studied when breathing 100% O_2_, and room air, hyperoxia had no effect at rest but attenuated the MSNA response to 50% dynamic handgrip (Stickland & Miller, 2008). In direct contrast, in our middle‐aged cohort, peripheral chemoreceptor inhibition by breathing 32% O_2_ reduced resting MSNA significantly, but had no impact on sympatho‐neural responses to 1‐leg cycling at either intensity. As HR responses were similar, reductions in MSNA burst incidence were also unaffected by hyperoxia. The present findings are consistent with those of hyperoxic studies by Seals et al. of healthy young men before and during 50% dynamic handgrip exercise (Seals et al., 1991).

The present protocol was designed to evaluate selectively, the impact of each of these reflexes on MSNA responses to 1‐leg cycling, rather than specific interactions. For example, in addition to noting reductions in resting HR, BP and MSNA in response to 100% oxygen, Houssiere and colleagues (Houssiere, Najem, Cuylits, et al., 2006), observed augmented BP and MSNA responses to 30% isometric handgrip plus PECO during hyperoxia, consistent with an interaction between the arterial chemoreflex and the muscle metaboreflex whereby the latter was sufficiently sensitized to over‐ride any sympatho‐inhibition consequent to concurrent interruption of peripheral chemoreflex input (Houssiere, Najem, Cuylits, et al., 2006). In young men, sympatho‐inhibition during mild cycling, supine, was reversed when peripheral chemoreceptors were exposed to a hypoxic stimulus (breathing 12.7% O_2_) (Katayama et al., 2011) or sympatho‐excitatory mechano‐ and metabo‐receptor afferents were stimulated further by higher exercise intensities (Katayama et al., 2020). As well, muscle metaboreflex activation can augment the MSNA response to peripheral chemoreceptor stimulation by hypoxia, without altering chemoreceptor sensitivity per se (Edgell & Stickland, 2014). Such interactions have yet to be characterized, similarly, in older age groups.

### 4.4 Reflex contributions to age‐related increases in MSNA

We recently reported an age‐related increase in MSNA at rest in a large cohort of healthy normotensive men and women (Keir et al., 2020). The present findings provide insight into potential reflex mechanisms contributing to this increase. In healthy young men, resting MSNA was lower and central venous pressure higher when recorded supine than sitting, a finding attributed to a shift in blood volume into the thorax, eliciting cardio‐pulmonary reflex‐mediated sympathetic inhibition (Ray et al., 1993). With respect to peripheral chemoreceptor reflexes, when MSNA was recorded from young volunteers during supine rest, firing frequency fell after they inhaled 100% O_2_ for 3–15 min, (Houssiere, Najem, Cuylits et al., 2006; Seals et al., 1991), but not after briefer exposure to hyperoxia (Stickland & Miller, 2008). In the present middle‐aged study participants, resting MSNA was inhibited by both a shift in blood volume after assuming the supine posture (4–6 bursts per minute, or 11%–15% on average) and also, and to a similar extent, by suppression of the peripheral chemoreflex by 32% O_2_. These changes are similar in magnitude to those observed after exercise training in patients with untreated hypertension (−5 bursts/min) (Ehlers et al., 2023) and heart failure (−6 bursts/min) (Notarius, Millar, Keir, et al., 2019), two diseases characterized by increased resting sympathetic drive. The present findings are consistent with the concept that unloading of augmented cardiopulmonary (Davy et al., 1998) and sensitization of peripheral chemoreceptor reflexes facilitate the sympathetic excitation of healthy aging.

### 4.5 Limitations

Because of the technical challenge of securing high quality and stable MSNA recordings from an exercising leg, we measured this in the contra‐lateral stationary leg and assumed that MSNA measured in the stationary leg was congruent. Our observations are limited to the exercise intensities and mode which we employed, however mild exercise intensity comprises the majority (65%–75%) of physical activity time spent as part of the activities of daily living in middle‐aged adults (Westerterp, 2013).

## 5 CONCLUSIONS

In summary, sympatho‐inhibition during mild but not moderate dynamic leg exercise in middle‐age reflects a robust cardiopulmonary baroreflex response. The muscle metaboreflex and arterial chemoreflex do not play a significant independent role in the sympathetic response during mild intensity dynamic leg exercise but reflex interactions, particularly at the moderate intensity cannot be ruled out. Interestingly, tonic activity of both the cardiopulmonary baroreflex, and arterial chemoreflex appear to modulate resting sympathetic nerve traffic in healthy middle‐age.

## AUTHOR CONTRIBUTIONS

John S. Floras and Catherine F. Notarius conceived and designed research, Catherine F. Notarius, Mark B. Badrov, Tomoyuki Tobushi, Daniel A. Keir, Evan Keys and John S. Floras performed experiments, Catherine F. Notarius analyzed data. Catherine F. Notarius, Mark B. Badrov, Evan Keys, and John S. Floras interpreted results of experiments. Catherine F. Notarius prepared Tables and Figures and drafted manuscript. All authors edited and revised manuscript drafts and approved final manuscript.

## FUNDING INFORMATION

This work was supported by operating grants from the Heart and Stroke Foundation of Ontario [grant numbers T4938, NA6298 to J.S.F.], the Canadian Institutes of Health Research [grant number PJT148836 to J.S.F.]. Daniel Keir and Mark Badrov were both recipients of a Post‐doctoral fellowship from the Canadian Institutes of Health Research. Dr. Floras held the Tier 1 Canada Research Chair in Integrative Cardiovascular Biology.

## CONFLICT OF INTEREST STATEMENT

The authors have no conflicts of interest to report.

## Data Availability

The data that support the findings of this study are available from the corresponding author upon reasonable request.
